# Permanent Pacing After Transcatheter Aortic Valve Implantation: Incidence, Predictors and Evolution of Left Ventricular Function

**DOI:** 10.5935/abc.20170170

**Published:** 2017-12

**Authors:** Cláudio Monteiro, Andres Di Leoni Ferrari, Paulo Ricardo Avancini Caramori, Luiz Antonio Ferreira Carvalho, Dimytri Alexandre de Alvim Siqueira, Luiz Eduardo Koenig São Thiago, Marco Perin, Valter C. de Lima, Enio Guérios, Fabio Sandoli De Brito Junior

**Affiliations:** 1 Centro de Pesquisas Cardiovasculares do Hospital São Lucas da PUCRS, Porto Alegre, RS - Brazil; 2 Hospital Pró-Cardíaco, Rio de Janeiro, RJ - Brazil; 3 Instituto Dante Pazzanese de Cardiologia, São Paulo, SP - Brazil; 4 SOS Cardio Serviços Hospitalares, Florianópolis, SC - Brazil; 5 Hospital Israelita Albert Einstein, São Paulo, SP - Brazil; 6 Santa Casa de Misericórdia de Porto Alegre, Porto Alegre, RS - Brazil; 7 Hospital de Clínicas da Universidade Federal do Paraná, Curitiba, PR - Brazil - Brazil

**Keywords:** Aortic Valve Stenosis, Atroventricular Block, Transcatheter Aortic Valve Replacement / complications, Pacemaker, Artificial, Stroke Volume

## Abstract

**Background:**

Transcatheter aortic valve implantation (TAVI) is a well-established procedure; however, atrioventricular block requiring permanent pacemaker implantation (PPI) is a common complication.

**Objectives:**

To determine the incidence, predictors and clinical outcomes of PPI after TAVI, focusing on how PPI affects left ventricular ejection fraction (LVEF) after TAVI.

**Methods:**

The Brazilian Multicenter TAVI Registry included 819 patients submitted to TAVI due to severe aortic stenosis from 22 centers from January/2008 to January/2015. After exclusions, the predictors of PPI were assessed in 670 patients by use of multivariate regression. Analysis of the ROC curve was used to measure the ability of the predictors; p < 0.05 was the significance level adopted.

**Results:**

Within 30 days from TAVI, 135 patients (20.1%) required PPI. Those patients were older (82.5 vs. 81.1 years; p = 0.047) and mainly of the male sex (59.3% vs 45%; p = 0.003). Hospital length of stay was longer in patients submitted to PPI (mean = 15.7 ± 25.7 vs. 11.8 ± 22.9 days; p < 0.001), but PPI affected neither all-cause death (26.7% vs. 25.6%; p = 0.80) nor cardiovascular death (14.1% vs. 14.8%; p = 0.84). By use of multivariate analysis, the previous presence of right bundle-branch block (RBBB) (OR, 6.19; 3.56-10.75; p ≤ 0.001), the use of CoreValve^®^ prosthesis (OR, 3.16; 1.74-5.72; p ≤ 0.001) and baseline transaortic gradient > 50 mm Hg (OR, 1.86; 1.08-3.2; p = 0.025) were predictors of PPI. The estimated risk of PPI ranged from 4%, when none of those predictors was present, to 63%, in the presence of all of them. The model showed good ability to predict the need for PPI: 0.69 (95%CI: 0.64 - 0.74) in the ROC curve. The substudy of 287 echocardiograms during the 1-year follow-up showed worse LVEF course in patients submitted to PPI (p = 0.01).

**Conclusion:**

BRD prévio, gradiente aórtico médio > 50 mmHg e CoreValve® são preditores independentes de implante de MPD pós-TAVI. Ocorreu implante de MPD em aproximadamente 20% dos casos de TAVI, o que prolongou a internação hospitalar, mas não afetou a mortalidade. O implante de MPD afetou negativamente a FEVE pós-TAVI.

## Introduction

Transcatheter aortic valve implantation (TAVI) is an alternative to conventional surgery for patients with severe aortic stenosis at high surgical risk.^1-3^ For more than one decade, that technology has proved to increase the quality of life and survival of patients, rapidly becoming a solid treatment option. Atrioventricular block (AVB) and the need for permanent pacemaker implantation (PPI) are complications commonly reported after surgical or percutaneous aortic valve replacement. The PPI rate after surgical aortic valve replacement has been recently reported as 5.8%,^4^ while that after TAVI ranges from 8% to 33.7%,^4,5^ according to the largest studies and meta-analyses. Previous publications of data from the Brazilian Multicenter TAVI Registry have reported an incidence of TAVI-related PPI around 25% in the first 30 days.^6^

The risk factors for the need for PPI remain inaccurate, being related to the characteristics of the patient (previous conduction system disease: right bundle-branch block - RBBB) and of the procedure, in which the intervention causes direct mechanical trauma, inflammation due to prosthesis positioning and balloon dilation,^4,7^ or even related to the device itself (self-expandable, balloon-expandable, tissue penetration). By analyzing data from the Brazilian Multicenter TAVI Registry, this study aimed at determining the incidence, predictors and clinical outcomes of PPI after TAVI, focusing on how PPI affects left ventricular ejection fraction (LVEF) after TAVI.

## Methods

### Study population

From January/2008 to January/2015, 819 patients submitted to TAVI with significant aortic valve stenosis, aortic valve area < 1 cm^2^ and mean transaortic gradient ≥ 40 mm Hg were included. After excluding those who died during the procedure, those who already had PPI and implantable cardioverter defibrillator, those who received an Inovare^®^ prosthesis, and those with unavailable or incomplete information about AVB prior to the intervention, 670 patients were left for analysis. The choice of the prosthesis was at the discretion of the operating physician. The indication for PPI was based on the institutional protocols of each participating hospital. The registry was approved by the Ethics Committee of all participating centers, and written informed consent was provided by all patients. Data were electronically monitored for identification and correction of inconsistent information. Local verification of the documents was randomly performed in 20% of all procedures.

### Evolution of LVEF

This study assessed the evolution of LVEF in a subgroup of 287 patients, whose echocardiographic data were available before the procedure and 1 year after that. In that subanalysis, clinical data related to the procedure and echocardiographic outcomes were compared between patients who underwent PPI within the first 30 days after TAVI and patients who did not. The outcome assessed was LVEF variation in 1 year, calculated according to the Simpson’s method.

### Statistical analysis

Atrioventricular block with subsequent PPI was attributed to TAVI when occurring within 30 days from that procedure. The patients were divided into two groups: "Group PPI", formed by patients who underwent PPI, and "Group non-PPI", formed by those who did not. Only two types of bioprostheses were included in the analysis: CoreValve^®^ (Medtronic Inc.; Minneapolis, MN, USA) and SapienXT^®^ (Edwards Lifesciences; Irvine, CA, USA). Categorical variables were presented as frequencies, being compared by using the chi-square or Fisher exact test. Continuous variables were presented as mean and standard deviation, being compared by using non-paired Student *t* test. The Kolmogorov-Smirnov test was used to assess if the quantitative variables had a normal distribution, and that supposition was confirmed.

Logistic regression was used to assess factors potentially associated with the need for PPI, with variables included in the model with level of significance ≤ 0.10. Multivariate regression analysis was performed adjusted for age, sex, pre- and post-dilation, heart rate before the procedure and presence of RBBB, and other types of intraventricular conduction disorders or the degree of AVB. Differences were statistically significant when p < 0.05. The ROC curves were analyzed to determine the ability of the risk factors to predict PPI. Outcomes within 30 days and 1 year were assessed with Kaplan-Meier curves and compared between the groups with the log-rank test. Predictors of LVEF change over time were analyzed with the use of a univariate and multivariate linear regression model. Statistical analysis was performed with the IBM-SPSS for Windows software, version 20.0.

## Results

From January/2008 to January/2015, data from 819 patients submitted to TAVI at 22 hospitals in Brazil were collected. Of those, 149 patients were excluded from the analysis due to: previous PPI or cardioverter defibrillator implantation (n = 86); incomplete or unavailable data about AVB prior to the intervention (n = 36); death during the procedure (n = 25); or Inovare^®^prosthesis implantation (Braile Biomedica; São José do Rio Preto, SP, Brazil; n = 20). Therefore, the study population was comprised of 670 patients as follows: Group PPI, formed by 135 patients (20.1%), and Group non-PPI, formed by 535 patients.

Table 1 lists the pre-procedure demographic and baseline clinical characteristics of the study population. Group PPI patients were slightly older (mean age, 82.5 ± 6.6 years *vs*. 81.1 ± 7,4 years; p = 0.047) and predominantly of the male sex (59.3% vs. 45%; p = 0.003). The risk scores (EuroScore I and Society of Thoracic Surgeons Score - STS) were similar between the groups. The presence of some degree of AVB on baseline electrocardiogram (ECG) increased the risk for need for PPI. It is worth noting that of the 135 patients requiring PPI, 36 (27.3%) had RBBB or RBBB associated with anterior hemiblock (AHB). That characteristic significantly predicted PPI after TAVI when compared to other conduction disorders (p ≤ 0.001).

**Table 1:** Pre-procedure demographic and clinical data of the population submitted to TAVI and its effect on permanent pacemaker implantation (PPI)

	PPI (n = 135)	Non-PPI (n = 535)	p value
Age (years)	82.5 ± 6.6	81.1 ± 7.4	0.047
Male sex	59.3% (80)	45.0% (241)	0.003
Systemic arterial hypertension	70.4% (95)	76.1% (407)	0.172
Dyslipidemia	48.9% (66)	48.6% (260)	0.952
Diabetes mellitus	34.8% (47)	31.6% (169)	0.474
Chronic kidney disease	71.1% (96)	76.8% (411)	0.167
Previous myocardial infarction	13.3% (18)	14.4% (77)	0.753
Previous TIA/stroke	9.6% (13)	8.0% (35)	0.550
Previous PCI	31.9% (43)	34.0% (182)	0.634
CABG	23.0% (31)	16.3% (87)	0.068
Peripheral vascular disease	13.3% (26)	15.9% (85)	0.346
Porcelain aorta	6.7% (9)	7.3% (39)	0.802
Pulmonary hypertension	17.8% (24)	21.3% (114)	0.365
COPD	22.2% (30)	18.3% (98)	0.302
Previous valvuloplasty	7.4% (10)	6.5% (35)	0.720
Previous valve replacement	1.5% (2)	4.5% (24)	0.106
Angina	29.6% (40)	22.1% (118)	0.064
Syncope	25.9% (35)	22.4% (120)	0.389
I or II	20.7% (28)	18.3% (98)	
III or IV	79.3% (107)	81.7% (437)	
EuroScore I	20.2 ± 15.3	20.1 ± 14.4	0.972
STS score	11.1 ± 8.4	10.2 ± 7.9	0.252
Creatinine clearance	49.3 ± 21.5	49.2 ± 22.1	0.951
**Heart rhythm**			**0.834**
Sinus	85.8% (115)	86.5% (462)	
Atrial fibrillation/flutter	14.2% (19)	13.5% (72)	
**Atrioventricular block**			0.045*
1^st^ degree	21.5 % (29)	14.0% (75)	
2^nd^ degree - Mobitz I	0.7% (1)	0% (0)	
2^nd^ degree - Mobitz II	0% (0)	0.2% (1)	
**Conduction disorder**			**< 0,001**
RBBB or RBBB+AHB	27.3% (36)	6.6% (35)	
LBBB	11.4% (15)	14.8% (78)	
AHB or none	61.4% (81)	78.6% (414)	

TIA: transient ischemic attack; PCI: percutaneous coronary intervention; CABG: coronary artery bypass grafting; CAD: coronary artery disease; COPD: chronic obstructive pulmonary disease; RBBB: right bundle branch block; LBBB: left bundle-branch block; AHB: anterior hemiblock.

(*) Likelihood ratio; Student t test for continuous variables; chi-square test for categorical variables.

Table 2 shows the pre-TAVI echocardiographic data. Group PPI patients had slightly higher mean aortic gradient (52.8 ± 16.0 mmHg *vs.* 49.5 ± 15.9 mmHg; p = 0.037) and thicker interventricular septum (12.7 ± 2.2 mmHg *vs*. 12.1 ± 2.2 mmHg; p = 0.013). There was no significant difference between the groups regarding pre-procedure LVEF (60.7% ± 12.1% in Group PPI vs. 59.0% ± 15.1% in Group non-PPI; p = 0.15).

**Table 2:** Baseline echocardiographic findings in patients with and without PPI after TAVI

	PPI (n = 135)	Non-PPI (n = 535)	p value
Aortic valve area (cm^2^)	0.65 ± 0.17	0.67 ± 0.20	0.427
Aortic valve ring (mm)	23.3 ± 3.1	22.9 ± 3.0	0.189
LVEF (%)	60.7 ± 12.1	59.0 ± 15.1	0.149
Peak gradient (mm Hg)	86.5 ± 26.2	81.5 ± 24.7	0.043
Mean gradient (mm Hg)	52.8 ± 16.0	49.5 ± 15.9	0.037
LV diastolic diameter (mm)	50.5 ± 9.0	50.6 ± 9.4	0.952
Septal thickness (mm)	12.7 ± 2.2	12.1 ± 2.2	0.013
LV posterior wall thickness (mm)	11.9 ± 2.4	11.6 ± 1.9	0.229
Aortic regurgitation	85.5% (112)	86.5% (453)	0.011*
Mild	76.3% (100)	71.8% (376)	
Moderate + Severe	9.2% (12)	14.7% (77)	
Mitral regurgitation	88.6% (117)	88.2% (463)	0.826*
Mild	72.7% (96)	69.9% (365)	
Moderate + Severe	15.9% (21)	18.8% (98)	

PPI: permanent pacemaker implantation; LVEF: left ventricular ejection fraction; LV: left ventricular.

(*) Likelihood ratio; Student t test for continuous variables.

Regarding the type of prosthesis, the need for PPI was more frequent in patients receiving the CoreValve^®^ prosthesis as compared to those receiving the Sapien^®^ device (23.9% *vs*. 9.3%, respectively; p ≤ 0.001). The other characteristics related to the procedure had no impact on the need for PPI (Table 3).

**Table 3:** Characteristics of the procedure in patients with and without PPI after TAVI

	PPI (n = 135)	Non-PPI (n = 535)	p value
**Anesthesia**			**0.769**
Sedation	8.9% (12)	9.7% (52)	
General	91.1% (123)	90.3% (483)	
**Vascular access**			**0.537**
Transfemoral or iliac	97.0% (131)	95.9% (513)	
Others	3.0% (4)	4.1% (12)	
Successful device implantation	88.9% (120)	89.2% (417)	0.928
Poor overlapping	3.7% (5)	4.5% (24)	0.690
Prosthesis migration or embolization	3.0% (4)	2.6% (14)	0.824*
Need for a second prosthesis	3.7% (5)	4.1% (22)	0.829
Transesophageal echocardiography	75.6% (102)	82.2% (440)	0.077
Pre-dilation	54.1% (73)	48.2% (258)	0.224
**Bioprosthesis type**			**< 0,001**
CoreValve	88.1% (119)	70.8% (379)	
SapienXT	11.9% (16)	29.2% (156)	
Post-dilation	40.7% (55)	37.0% (198)	0.424

(*) Likelihood ratio; Student t test for continuous variables; chi-square test for categorical variables.

### Predictors of PPI

The multivariate analysis (Table 4), describing the independent risk factors for PPI within 30 days after TAVI, confirmed RBBB alone or in association with AHB as a strong risk factor (OR 6.19; 95%CI: 3.56-10.76; p < 0.001), as well as the CoreValve^®^ device (OR 3.16; 95%CI: 1.74-5.72; p < 0.001). In addition, mean transaortic gradient (OR 1.86; 95%CI: 1.08-3.20; p = 0.025), the innovative finding of this study, was an independent predictor of the need for PPI. Table 5 shows the likelihood of the need for PPI estimated by multiple logistic regression combining the independent predictors of PPI within 30 days after TAVI. To build the model, the mean transaortic gradient value was analyzed as a categorical variable, using the cutoff point of 50.05 mmHg, determined based on the mean of the total population of the registry.

**Table 4:** Independent predictors of the need for PPI after TAVI

Variable	OR (95%CI)	p value
**Conduction disorder**		
RBBB or RBBB+AHB	6.19 (3.56-10.76)	< 0.001
**Bioprosthesis type**		
CoreValve	3.16 (1.74-5.72)	< 0.001
**Mean gradient**		
≥ 50 mm Hg	1.86 (1.08-3.20)	0.025

RBBB: right bundle-branch block; AHB: anterior hemiblock; the mean transaortic gradient was the mean found in the population: 50.05 mm Hg. Multiple logistic regression.

**Table 5:** Likelihood of PPI within the first 30 days after TAVI according to 3 independent variables on multivariate analysis

Conduction disorder		Bioprosthesis type		Mean gradient		PPI likelihood (%) within 30 days
AHB or LBBB	RBBB or RBBB+AHB	CoreValve	SapienXT	< 50	≥ 50	
X			X	X		4.4
X			X		X	8.0
X		X		X		12.8
X		X			X	21.5
	X		X	X		22.4
	X		X		X	34.9
	X	X		X		47.6
	X	X			X	62.9

PPI: permanent pacemaker implantation; AHB: anterior hemiblock; LBBB: left bundle-branch block; RBBB: right bundle-branch block.

### Impact of PPI on hospitalization, clinical outcomes and LVEF

The hospital length of stay in the Group PPI was significantly prolonged (mean = 15.7 ± 25.7 days - Group PPI vs. 11.8 ± 22.9 days - Group non-PPI; p < 0.001). No difference was observed between the groups regarding all-cause mortality (26.7% vs. 25.6% for groups PPI and non-PPI, respectively; p = 0.80) and cardiovascular mortality (14.1% vs. 14.8% for groups PPI and non-PPI, respectively; p = 0.84) during hospitalization.

In the substudy of 287 patients with echocardiograms before the procedure and 1 year after that, 74 patients received PPI. The groups did not differ regarding baseline LVEF (Group PPI: 60.7% ± 12.1% vs. Group non-PPI: 59.0% ± 15.1%; p = 0.15), but differed significantly regarding the 1-year follow-up after TAVI (mean variation: -2.27% ± 13.46 for Group PPI vs. 3.28% ± 11.99 for Group non-PPI; p = 0.01). Baseline LVEF and need for PPI within 30 days after TAVI were the only independent predictors of LVEF worsening over time (estimated coefficient -0.51; 95%CI: -0.62 to -0.40; p < 0.001; and -4.92; 95%CI: -8.14 to -1.69; p = 0.003, R^2^= 0.35, respectively; Table 6). That negative association of PPI with LVEF had no impact on the NYHA functional class (p = 0.35 on multivariate analysis).

**Table 6:** Univariate and multivariate predictors of changes in left ventricular ejection fraction over time (12 month follow-up)

	Univariate		Multivariate	
	Coefficient (95% CI)	p value	Coefficient (95% CI)	p value
**Clinical variables**				
Age	-0.043 (-0.259 to 0.173)	0.699		
Sex	0.179 (-2.89 to 3.252)	0.909		
Hypertension	-3.673 (-6.938 to -0.408)	0.318	-0.667 (-3.548 to 2.214)	0.650
Diabetes mellitus	-1.753 (-5.187 to 1.681)	0.318		
eGFR < 60 mL/min	1.475 (-2.253 to 5.203)	0.439		
Paroxysmal/chronic atrial fibrillation	1.937 (-2.828 to 6.702)	0.426		
Coronary artery disease	0.274 (-2.801 to 3.349)	0.861		
Echocardiography			-0.511 (-0.619 to -0.403)	
LVEF	-0.466 (-0.554 to -0.378)	<0.001	0.033 (-0.061 to 0.127)	<0.001
Mean gradient (≥ 50.05 mm Hg)	-0.143 (-0.24 to -0.043)	0.006		0.491
Aortic valve area	-0.216 (-8.227 to 7.795)	0.958	-0.131 (-0.286 to 0.024)	
LV diastolic diameter	0.166 (-0.001 to 0.333)	0.053		0.098
**Variables of the procedure**				
Moderate or greater AR	-0.085 (-4.595 to 4.425)	0.971	-4.917 (-8.141 to -1.693)	
Within 30 days from PPI	-5.55 (-9.221 to -1.879)	0.003		0.003
CoreValve	-0.708 (-4.577 to 3.161)	0.720		
Pre-dilation	-2.516 (-5.648 to 0.616)	0.117	1.652 (-1.772 to 5.076)	
HF (III or IV)	5.578 (1.676 to 9.480)	0.005		0.345

AR: aortic regurgitation; CI: confidence interval; eGFR: estimated glomerular filtration rate; LVEF: left ventricular ejection fraction; LV: left ventricular; PPI: permanent pacemaker implantation; HF: heart failure. Linear regression; multivariate model R^2^ = 0.347.

**Figure 1:**
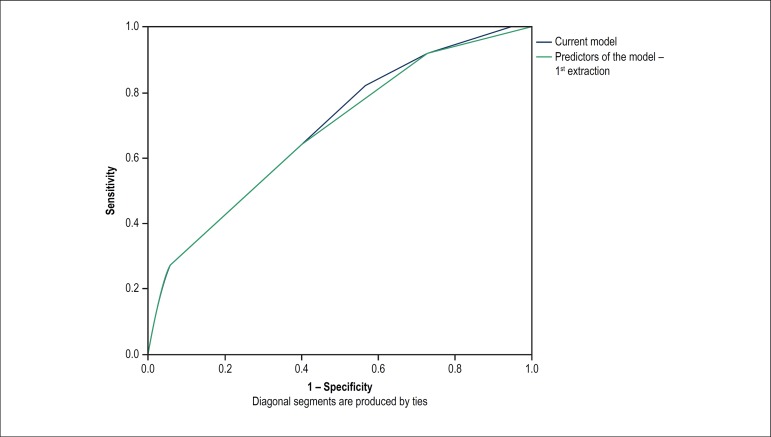
ROC curve comparing the performance of the predictors previously published by the Brazilian Multicenter TAVI Registry and the new ones

The area under the ROC curve for the model of predictors (Figure 1) showed good competence to predict the need for PPI: 0.69 (95%CI: 0.64 - 0.74).

## Discussion

Transcatheter aortic valve implantation has been established not only as an effective treatment for patients for whom conventional surgery is not an option, but also as an alternative to patients at high^8^ and, more recently, moderate risk. The need for PPI due to total AVB is a frequent complication of TAVI. Under other clinical circumstances, PPI has been associated with left ventricular systolic function impairment, possibly secondary to the negative impact of PPI on LVEF due to the dyssynchrony inflicted by the artificial electromechanical activation on left ventricular performance.^9^ The major findings of this study are the description of the predictors of need for post-TAVI PPI in the Brazilian population and the description of the unfavorable effect of PPI on LVEF by the end of the first year after the implantation.

The native aortic valve apparatus lies very close to the AV node and His bundle, therefore, TAVI might harm the infra-Hisian conduction system, probably due to direct pressure and compression, hemorrhage/hematoma, ischemia or inflammation of the His bundle and compact AV node during the prosthesis positioning or expansion.^4,7,10-12^ Thus, heart block can occur early after TAVI. The Valve Academic Research Consortium (VARC) has highlighted the risk of AVB requiring PPI as one of the most relevant complications associated with TAVI.^10,13-17^ However, TAVI has been shown to improve the left ventricular systolic function,^18^ but patients requiring PPI might fail to recover as expected due to the right ventricular stimulus, unfavorable to left ventricular systolic performance.^4,9,18-21^

In this study population, considering a pre-TAVI LVEF similar in both groups and adjusting for clinical, echocardiographic and procedural variables, the patients submitted to post-TAVI PPI showed a significantly reduced LVEF by the end of the first year. In fact, PPI within the first 30 days after TAVI and baseline LVEF were the only factors that significantly worsened left ventricular performance (approximately 6%) in that period. Such data are in accordance with previously published reports.^19,21^ However, that is not a consensus and has been recently challenged by the findings of other studies,^4,20^ showing that the issue requires further consideration. However, from the clinical perspective, in our substudy, the negative association of PPI with LVEF had no impact on the NYHA functional class of heart failure. This can be partially explained by the fact that the baseline LVEF was normal in most of the population, because of the small deterioration of LVEF observed in most patients and because of the positive hemodynamic effects related to aortic stenosis repair.

The major findings of the analysis of the risk factors for the need for PPI after TAVI were: 1. One PPI for every five TAVI performed (20.1%); 2. Previous RBBB (isolated or associated with AHB), mean transaortic gradient and use of CoreValve^®^ bioprosthesis were independent predictors of PPI; and 3. The likelihood of PPI after TAVI ranges from 4.4%, when none of those risk factors are present, to 62.9%, in the presence of those three.

The proportion of patients from the Brazilian Multicenter TAVI Registry requiring PPI after TAVI is in accordance with data from European countries (16.3% in the UK TAVI Registry,^22^ and 13% in the Belgian National Registry^23^). However, that is approximately half of the 33.7% incidence observed in the German TAVI Registry.^24,25^ In a recent meta-analysis,^26^ comprising more than 11000 patients, 17% of them required PPI after TAVI. In another systematic review^27^ with more than 2000 patients from European and North American retrospective studies, the incidence of PPI after TAVI was 14.2% (ranging from 0 to 34%; median of 9.7%).

The indication for PPI and its time of performance are frequently individualized according to the center and/or the operating physician’s preference. The current European Society of Cardiology guidelines^28^ on cardiac pacing and cardiac resynchronization therapy recommend, regarding AVB after TAVI, PPI be performed before completing the observation period of 7 days only if the escape rhythm is considered low or unstable (class of recommendation I, level of evidence C).

The finding that PPI prolongs the hospital length of stay is no surprise, being in accordance with previous studies.^4,21,29,30^ Although this study does not assess costs, the need for PPI is intuitively associated with an increased use of hospital resources and might have resulted in a considerable increase in the general costs of TAVI. In addition, PPI requires an additional surgical procedure that is not risk-free. However, in accordance with previous publications,^21^ our data show that PPI influences neither global mortality nor cardiovascular mortality.

The reported predictors of PPI after TAVI have shown some variability and heterogeneity between the publications, ^4,6,18,20,21,26,29-33^ indicating that the mechanism associated with AVB could be multifactorial. Being a factor related to the patient, the conduction disorders have been consistently reported in the literature, but with different importance. While the predictive role of RBBB has been accepted, the meaning of developing left bundle-branch block (LBBB), a common disorder after TAVI, is still uncertain.^1,34,35^ Likewise, the influence of age and the differences related to sex remain controversial. Some anatomical and echocardiographic characteristics, such as septal wall dimensions, non-coronary cusp thickness, porcelain aorta, aortic subvalvular calcification, valvular ring diameter, have been reported. This analysis of the Brazilian Multicenter TAVI Registry failed to show an association of those characteristics with the need for PPI. However, we found a new independent predictor associated with the likelihood of PPI after TAVI, the mean transaortic gradient. We interpreted that as representing the greater severity of the valvular apparatus calcification. There is neither a study nor a registry investigating directly the effects of that echocardiographic parameter or its influence as a predictor of the need for PPI. Therefore, that finding might have a speculative importance, requiring further investigation.

Regarding the aspects related to the device, there are differences in composition and design, delivery mechanism and tissue penetration ability. In this study, the need for PPI among patients receiving the SapienXT^®^ device (Edwards Lifesciences; Irvine, CA, USA) is very close to that reported in the literature^4,28^(5.9% - 6.5%). In addition, the PPI rates related to CoreValve^®^ implantation (Medtronic Inc.; Minneapolis, MN, USA) are known to be substantially greater and in accordance with recent publications^4,26^ (24.5% - 25.8%).

Finally, our data are in accordance with those of most studies and registries, in which previous RBBB (isolated or associated with AHB) and the CoreValve^®^prosthesis type are almost unanimously accepted as independent predictors of the risk for requiring PPI after TAVI.^18,20,21,26,31,33,34,36^

### Study limitations

This is an analysis from a non-randomized registry, of voluntary participation, which has inherent restrictions, associated with the limitations of retrospective data analysis, issues related to the uniformity of patient selection process and outcome description. This registry represents neither all centers nor the total number of TAVI performed in Brazil. Furthermore, it does not include all devices available for TAVI in the Brazilian market, contemplating only two types of bioprostheses internationally implanted. The PPI was performed at the discretion of the participating centers and the registry had no information on that procedure, and the following aspects could not be assessed: stimulation site, QRS duration, and AVB reversibility potential (up to 50% in some publications^1,27,37-40^). Finally, the echocardiographic data before the procedure and 1 year after it were available in approximately half of the population (287 patients). The LVEF was reported by each participating center, which can add more variability to the findings.

## Conclusion

**Figure 2:**
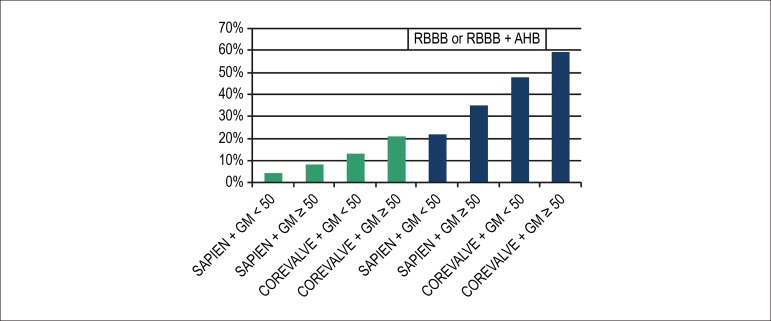
Risk model: likelihood of permanent pacemaker implantation within 30 days after TAVI based on predictors of the Brazilian Multicenter TAVI Registry. RBBB: right bundle-branch block; AHB: anterior hemiblock; the mean transaortic gradient was the mean found in the population: 50.05 mm Hg

Permanent pacemaker implantation is the most frequent post-TAVI complication, and its consequences extend beyond the surgical procedure inherent in implantation. In this analysis of the Brazilian Multicenter TAVI Registry, the need for PPI after TAVI is a relatively frequent finding (incidence of 20.1%), and PPI can have adverse effects, such as worse LVEF recovery. In addition, the need for PPI prolonged the post-procedure hospital length of stay, but was not associated with global mortality, cardiovascular death or heart failure functional class worsening. In accordance with previous reports, RBBB (isolated or associated with AHB) and the use of CoreValve^®^prosthesis were important predictors of the need for PPI after TAVI. In addition, this study identified pre-procedure mean transaortic gradient as a new risk factor. A simple model of predictors (Figure 2) was elaborated to estimate the absolute risk of PPI after TAVI in the Brazilian population. These risk factors can be used to identify individuals at high risk for PPI, which can be a useful tool for resource planning.
